# Lp(a) in the Pathogenesis of Aortic Stenosis and Approach to Therapy with Antisense Oligonucleotides or Short Interfering RNA

**DOI:** 10.3390/ijms241914939

**Published:** 2023-10-06

**Authors:** Assunta Di Costanzo, Ciro Indolfi, Anna Franzone, Giovanni Esposito, Carmen Anna Maria Spaccarotella

**Affiliations:** 1 Division of Cardiology, Cardiovascular Research Center, University Magna Graecia Catanzaro, 88100 Catanzaro, Italy; indolfi@unicz.it; 2 Division of Cardiology, Department of Advanced Biomedical Sciences, University of Naples Federico II, 80131 Naples, Italy; anna.franzone@unina.it (A.F.); giovanni.esposito2@unina.it (G.E.); carmenannamaria.spaccarotella@unina.it (C.A.M.S.)

**Keywords:** Lp(a), dyslipidemia, inflammation, calcification, aortic valve stenosis, TAVI, AVR, pelacarsen, olpasiran, artificial intelligence

## Abstract

To date, no medical therapy can slow the progression of aortic stenosis. Fibrocalcific stenosis is the most frequent form in the general population and affects about 6% of the elderly population. Over the years, diagnosis has evolved thanks to echocardiography and computed tomography assessments. The application of artificial intelligence to electrocardiography could further implement early diagnosis. Patients with severe aortic stenosis, especially symptomatic patients, have valve repair as their only therapeutic option by surgical or percutaneous technique (TAVI). The discovery that the pathogenetic mechanism of aortic stenosis is similar to the atherosclerosis process has made it possible to evaluate the hypothesis of medical therapy for aortic stenosis. Several drugs have been tested to reduce low-density lipoprotein (LDL) and lipoprotein(a) (Lp(a)) levels, inflammation, and calcification. The Proprotein Convertase Subtilisin/Kexin type 9 inhibitors (PCSK9-i) could decrease the progression of aortic stenosis and the requirement for valve implantation. Great interest is related to circulating Lp(a) levels as causally linked to degenerative aortic stenosis. New therapies with ASO (antisense oligonucleotides) and siRNA (small interfering RNA) are currently being tested. Olpasiran and pelacarsen reduce circulating Lp(a) levels by 85–90%. Phase 3 studies are underway to evaluate the effect of these drugs on cardiovascular events (cardiovascular death, non-fatal myocardial injury, and non-fatal stroke) in patients with elevated Lp(a) and CVD (cardiovascular diseases). For instance, if a reduction in Lp(a) levels is associated with aortic stenosis prevention or progression, further prospective clinical trials are warranted to confirm this observation in this high-risk population.

## 1. Introduction

**Figure 1:**
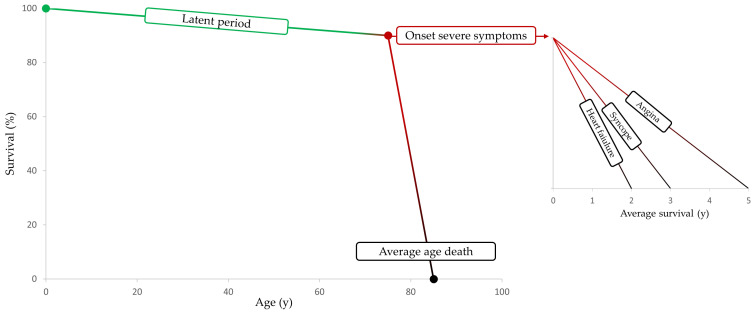
Prognosis of symptomatic patients with severe aortic stenosis in the absence of specific treatment (modified from Ross and Braunwald [4]). y, years.

Aortic valve stenosis (AVS) is the most frequent cardiac valve disease in the general population (0.4%), affecting 2% of subjects aged 65 and 12% of patients aged greater than 75 [1]. The degenerative/calcific (or senile) cause is the most common type of aortic stenosis in the elderly population; its incidence increases with age and is due to the establishment of fibrocalcific processes [2]. The prognosis is unfavorable from the onset of typical symptoms: angina, syncope, and heart failure. In the absence of specific treatment, the onset of symptoms is associated with a survival rate of 50% at a 2-year follow-up [3] (Figure 1).

The history of aortic stenosis has radically evolved in recent decades. Initially, the pathological process was thought to be related to wear and tear. However, it has been shown that the mechanisms responsible for aortic stenosis are processes that are more similar to atherosclerosis. The pathogenesis is linked to an activity involving genetic mechanisms, lipoproteins, inflammation, and mineralization of the valve leaflets [5,6]. The process results in valve stenosis with obstruction of flow from the left ventricle to the systemic circulation [7]. The advent of echocardiography has revolutionized the diagnosis and quantification of AVS [8]. Recently, the novelty of artificial intelligence has made it possible to correlate electrocardiographic changes to a cluster of patients with overt aortic stenosis or predisposition to valve disease [9].

The only effective therapy for severe aortic stenosis is valve replacement by surgery or percutaneous treatment valve implantation. Transcatheter aortic valve implantation (TAVI) has allowed elderly patients or those judged inoperable to be treated. The ESC guidelines recommend TAVI in class I level of evidence A for all patients over 75 years [10]. The ACC/AHA guidelines go even further and recommend TAVI in class I for patients over 65 years according to a shared decision with the heart team [11]. However, although TAVI has become a routine procedure in cath labs and operators are increasingly experienced, periprocedural complications still exist. Major risks are bleeding complications, stroke, atrioventricular blocks requiring a pacemaker, and death [12,13].

No therapy has been clearly shown to slow or stop aortic valve disease progression, and understanding the pathophysiology of AVS may lead to pharmacological treatment.

## 2. Pathogenesis of Aortic Stenosis: Lipids, Inflammation, and Mineralization

The aortic valve is composed of three leaflets with a trilaminar structure. The outer layer, facing the aorta, is “fibrous” and composed of circumferentially oriented type I and III collagen fibers. The outer layer, directed toward the left ventricle, consists of radially oriented elastic fibers. These fibers allow this layer to expand under pressure and have greater compliance. These two layers are covered by endothelial cells that act as an interface between the blood and the valve. The inner layer comprises “spongiosa”; the most represented cells are the valve interstitial cells (VICs) [14]. During valvulogenesis, VICs are localized on the surface of the leaflet and have an endothelial origin. Subsequently, these cells migrate into the inner layer, become multipotent mesenchymal stem cells, and remain in the matrix as interstitial cells [15]. VICs are responsible for the phenotypic switch to osteogenic cells. The reprogramming of VICs underlies the mineralization of the valve and the progression to calcific stenosis [16].

**Figure 2:**
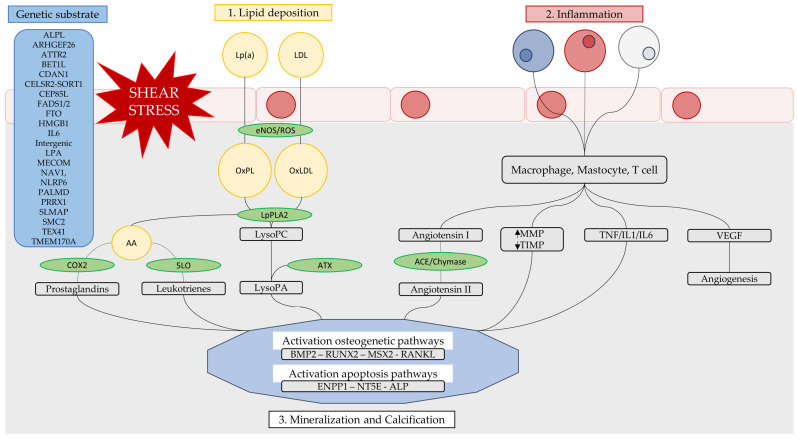
The pathogenetic mechanism of aortic valve stenosis: dyslipidemia, inflammation, and calcification. The figure summarizes the main pathways involved in the aortic valve calcification process. Lp(a), lipoprotein(a); LDL, low-density lipoprotein; eNOS, endothelial nitric oxide synthase; ROS, reactive oxygen species; OxPL, oxidized phospholipids; OxLDL, oxidized LDL; LpPLA2, lipoprotein-associated phospholipase A2; LysoPC, lysophosphatidylcholine; ATX, autotaxin; LysoPA, lysophosphatidic acid; AA, arachidonic acid; COX2, cyclooxygenase-2; 5LO, 5-lipoxygenase; MMP, matrix metalloproteinases; TIMP, tissue inhibitor of metalloprotease; TNF, tumor necrosis factor; IL1, interleukin-1; IL6, interleukin-6; VEGF, vascular endothelial growth factor; ACE, angiotensin converting enzyme; BMP2, bone morphogenetic protein 2; RUNX2, runt-related transcription factor 2; MSX2, Msh Homeobox 2; RANKL, receptor activator of nuclear factor κB Ligand; ENPP1, ectonucleotide pyrophosphatase/phosphodiesterase I; NT5E, ecto-5′-nucleotidase; ALP, alkaline phosphatase.

Initially, the pathological process was thought to be related to “wear and tear”. Today, it is thought to be a process more similar to atherosclerosis in subjects with a predisposing genetic substrate. The calcification process has a first phase of lipid deposition and inflammatory infiltration and a second phase of pro-calcific and pro-osteogenic factors activation [17] (Figure 2).

Genetic polymorphisms are associated with this pathology [18,19,20,21]. Recently, two genome-wide association studies (GWAS) analyzed numerous genetic variants in many genomes to find those statistically associated with calcific severe aortic stenosis. Chen et al. recently performed a meta-analysis of the entire genome of 653,867 participants, recognizing 17 loci associated with AVS, of which only seven were previously known (ALPL, PALMD, NAV1, TEX41, LPA, IL6, and FADS1/2) and 10 were completely new (CELSR2-SORT1, PRRX1, ATTR2, ARHGEF26, SMC2, BET1L, NLRP6, HMGB1, TMEM170A, and Intergenic) [22]. Small et al. recognized six new loci for calcific aortic stenosis (CEP85L, FTO, SLMAP, CELSR2, MECOM, and CDAN1) [23]. These meta-analyses also correlated the incidence of aortic stenosis with risk factors such as inflammation, calcification, adiposity, and dyslipidemia, particularly with lipoprotein(a) and low-density lipoprotein cholesterol blood levels.

Fibrosis and calcification are the processes that modify the biomechanical properties of valve leaflets. The initial or preclinical lesion is represented by aortic sclerosis, which affects ¼ of patients over 65 years and half of patients over 85 years [24]. The initial lesion is the focal calcifications and leaflet thickening without alterations in gradients and velocity. The primum movens is endothelial damage, or “shear stress”, resulting in lipid infiltration, particularly LDL and Lpa [25,26,27,28,29]. It has been assessed that the levels of apoB, apoE, apoA1, and apo(a) are present in surgically removed stenotic aortic valves [30]. The activation of the inflammatory process causes dysregulation of the eNOS (endothelial nitric oxide synthase) pathway and ROS production [31]. This initiates lipid oxidation with the transformation of Lpa into Ox-LP and LDL into OxLDL [32]. The lipids accumulated in the valve carry enzymes: LpPLA2 transported by LDL and autotaxin (ATX) transported by Lpa [33,34,35,36]. These enzymes produce lysophospholipid derivatives, bioactive lipid compounds. LpPLA2 converts Ox-LP into lysophosphatidylcholine (LysoPC), which causes the activation of the apoptotic process of VICs [37]. In addition, LpPLA2 generates AA (arachidonic acid) that promotes the formation of inflammatory molecules via the cyclooxygenase2 (Cox2) and 5-lipoxygenase (5-LO) pathways [38]. These proinflammatory molecules promote mineralization by increasing the expression of bone morphogenetic proteins 2 and 6 (BMP2 and BMP6). A mouse study showed that loss of Cox2 function reduces valve mineralization [39,40]. The autotaxin converts LysoPC into lysophosphatidic acid (LysoPA) [41]. After binding the LPAR receptor, LysoPA activates the NfkB pathway (RANKL) and Wnis t3a is involved in the osteogenetic transition of VICs. In vitro, the absence of autotaxin prevented mineralization. In vivo models, the administration of LysoPA increased aortic valve mineralization. Accordingly, the correlation between autotaxin, LysoPA, and mineralization explains the correlation between aortic stenosis and Lpa [42]. Oxidized lipids activate the immune response via the NfkB pathway and TLRs, which are also expressed by VICs (TLR 2 and 4) [43,44].

The inflammatory infiltrate consists of macrophages, monocytes, mast cells, and T cells that play an important role in remodeling the leaflets [45,46,47]. Interestingly, ACE (angiotensin-converting enzyme) and chymase are overexpressed in calcific aortic valves [48]. Mast cells secrete chymase, while ACE is carried by LDL [49]. ACE converts angiotensin I into angiotensin II. Angiotensin II is a potent activator of NfkB, and this also explains the high levels of IL6 and TNFa in the VICs. This pathway stimulates collagen production by VICs. The administration of olmesartan (ARB) in an animal model prevents aortic valve thickening [50,51].

Macrophages secrete VEGF, MMPs, TNFa, and IL1. The increased production of MMPs and reduced production of TIMPs allows the accumulation of collagen (secreted by VICs) in the aortic valve and the formation of disorganized fibrous tissue. The secretion of VEFG and neo-angiogenesis has a role that is still not completely known, but they participate in the recruitment of inflammatory and osteoprogenitor cells [52]. 

TNFa and IL1b activate the NfkB pathway resulting in increased IL6 concentration. These cytokines and oxidized lipids promote mineralization and osteogenesis by activating osteogenetic transduction pathways that promote mineralization: BMP2, RUNX2, and MSX2, and RANKL [53]. BMP2 promotes the expression of MSX2 and RUNX2 (bone-related transcription factors), positive regulators of the Wnt pathway. Wnt ligand (Wnt3a) is overexpressed in stenotic valves and plays a role in stabilizing b-catenin, a key factor in the osteogenetic differentiation of VICs. RANKL binds its receptor RANK by increasing the production of extracellular matrix by VICs. In vivo, experiments showed that the administration of osteoprotegerin blocks mineralization by inhibiting RANKL/RANK binding. 

Mineralization is associated with a joint process of osteogenesis and apoptosis. The VIC apoptosis process is triggered by ROS, cytokines, and alterations in the purinergic signaling pathway [54]. ATP prevents mineralization by binding to the purine receptor P2Y2. P2Y2 prevents apoptosis and increases the production of carbonic anhydrase 12 (CA12). CA12 acidifies the extracellular space and prevents deposits of amorphous calcium and phosphorous crystals [55]. The osteogenesis process is triggered by the activation of ENPP1, NT5E, and ALP pathways. ENPP1 hydrolyses ATP into AMP and PPi. ALP converts PPi into Pi; PPi has an inhibitory action on mineralization; on the contrary, Pi is a powerful inducer of mineralization. NT5E converts AMP into adenosine and Pi. This reduces ATP levels and increases Pi levels with the formation of calcium- and phosphorus-rich vesicles. Subsequently, the apoptotic VIC bodies become nests for the deposition of amorphous calcium and phosphorous crystals. 

Normally, the process of valve osteogenesis is prevented because the endothelium of the aortic valve expresses several anti-osteogenic genes (chordin and OPG). The cause of the switch is shearing stress and dysregulation of regulatory genes on the aortic side. The pathology is side-dependent, relative to different hemodynamic conditions. The ventricular side of the valve is exposed to unidirectional, pulsatile shear stress. The aortic side of the valve is more exposed to oscillatory shear stress and is correlated with increased expression of inflammatory markers (such as VCAM-1, ICAM-1, TGFβ-1, and BMP-4), miRNAs, and disease. The cause could be a different expression of regulatory genes, lower expression of negative regulators, higher expression of miRNAs, and mRNAs capable of inducing disease progression [56,57]. Several miRNAs have been associated with the development of aortic stenosis. In particular, protective molecules include miR-141, which blocks the BMP2 pathway; on the contrary, miR-34a promotes osteogenesis by the Notch1-Runx2 pathway, miR-486 activates the osteogenic Akt pathway, and miR-137 and miR-155 are involved in the activation of mineralization. Inhibition of these miRNAs could be a therapeutic strategy [58]. One of the regulators is NOTCH1, which negatively regulates the expression of the RUNX2 and BMP2 genes. Normally, NOTCH1 responds to shear stress by activating anti-calcific genes. N1 haploinsufficiency has been shown to activate pro-calcific pathways, leading to the dysregulation of about 1000 genes involved in osteogenesis, inflammation, and oxidative stress [59]. 

In summary, studies conducted in recent years have shown that the pathogenetic process is linked to lipid infiltration, inflammation, and osteogenetic response. Endothelial damage is the event that triggers lipid infiltration into the valve. These generate various bioactive lipid species that attract the mediators of inflammation. The activation of different signal transduction pathways causes dysregulation of VICs with a switch to an osteogenetic phenotype that promotes fibrocalcific remodeling of aortic valve leaflets.

## 3. Diagnosis and Treatment of Aortic Stenosis

Transthoracic Echocardiography (ETT) is the gold standard for the diagnosis and follow-up of aortic stenosis [60] (Table 1).

In the presence of a reduced ejection fraction, a functional study with the pharmacological stressor (echo-dobutamine) is required to define the severity of the valve defect and ventricular contractile reserve (SVi). Furthermore, the introduction of STE (speckle tracking echocardiography) has made it possible to assess changes in myocardial work [62,63].

The advent of artificial intelligence may be changing the screening and diagnosis of aortic stenosis [64]. Cohen Shelly et al. demonstrated that an ECG, a simple routine test, can recognize moderate-to-severe aortic stenosis (AUC 0.85) in asymptomatic subjects with high sensitivity and specificity, but above all, a high negative predictive value (VPN 99%) [65]. Diagnosis in asymptomatic patients may be useful to identify patients with cardiac maladaptation to AVS, to reduce sudden death, which affects 4.1% of patients with severe asymptomatic aortic stenosis, and refer patients earlier to AVR [66]. A recent clinical trial demonstrated a better clinical outcome in the early correction of the valve defect [67]. 

Cardiac Computed Tomography (CCT) provides relevant information on aortic valve anatomy, annulus size and shape, extent, and distribution of calcifications, risk of coronary ostial obstruction, aortic root size, and feasibility of vascular accesses [68]. 

**Figure 3:**
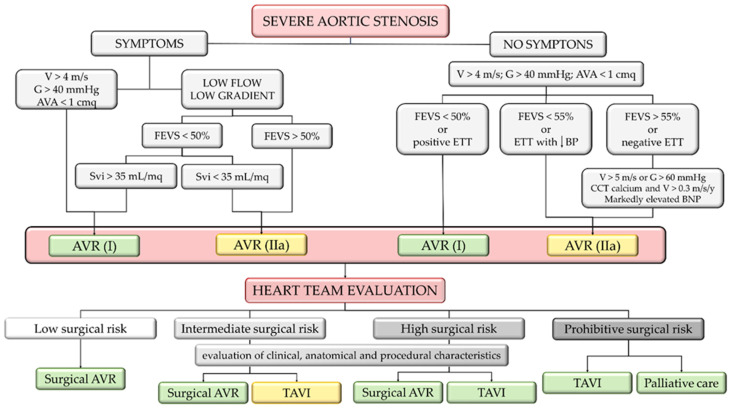
Flow chart for severe aortic stenosis treatment, according to VHD ESC guidelines 2021. In the figure, the green boxes indicate class I of recommendation, and the yellow boxes indicate class IIa of recommendation. The red box groups all conditions that need to be assessed by the heart team. V, aortic valve peak velocity; G, aortic valve mean gradient; AVA, aortic valve area; LVEF, left ventricular ejection fraction; Svi, systolic volume index; ETT, exercise tolerance test; BP, blood pressure; CCT, Cardiac Computed Tomography; BNP, brain natriuretic peptide; AVR, aortic valve implantation; TAVI, transcatheter aortic valve replacement.

Currently, no medical therapy influences the natural history of AVS. Therefore, aortic prosthesis implantation remains the only available therapy for severe symptomatic aortic stenosis. Recommendations for the indication and timing of surgery in patients with aortic stenosis are described in the AHA/ACC 2020 and ESC 2021 guidelines (Figure 3).

In recent years, the TAVI technique has evolved with the increased experience of operators, the use of ultrasound-guided cannulation, and the introduction of new valves, and today, TAVI is considered a routine therapeutic strategy in many laboratories [69,70]. EVOLUTE LOW-RISK and PARTNER-3 studies have shown that TAVI is not inferior to SAVR, even in low-risk patients [71,72,73]. However, TAVI is associated with risks such as death, bleeding complications, atrioventricular blocks requiring a pacemaker, and stroke [74,75,76].

Medical therapy could be useful to slow or stop the progression of the valvular disease and avoid or procrastinate surgery for patients.

## 4. Drug Therapy Approach to Aortic Stenosis

Currently, there is no approved drug therapy for degenerative aortic stenosis. The target mechanisms of new therapies are essentially dyslipidemia, in particular circulating Lp(a) levels, inflammation, and calcification.

Bisphosphonates reduce the circulating availability of procalcifying substances (calcium and phosphate) and exert an anti-calcifying action on aortic tissue. The action at the valve level is related to the reduction in proinflammatory cytokines (IL1, IL6, and TNFa), metalloprotease inhibition, and VICs differentiation. The MESA meta-analysis described less valve calcification associated with the use of bisphosphonates [77]. At present, the results are mixed, and a randomized controlled clinical trial will be necessary [78,79].

Denosumab is a monoclonal antibody that acts on the OPG/RANK/RANKL pathway. Specifically, through binding RANKL, it blocks the ligand–receptor interaction. Consequently, it blocks the osteogenic pathway, increases circulating OPG levels, and reduces the circulating calcium and phosphate levels. Promising results were obtained in a mouse model. Helas et al. described a halving of cases of aortic valve calcification [80]. In randomized trials, both denosumab and alendronic acid did not influence the progression of aortic valve calcification [81].

The ADVANCE study described a reduction in valve calcifications in nephropathic patients affected by secondary hyperparathyroidism treated with cinacalcet. Cinacalcet is a calcium receptor agonist used to control calcium and phosphate values in dialysis patients with secondary hyperparathyroidism. The limitation is its use only in dialytic patients [82].

Several studies have described the association between warfarin and the development or progression of aortic stenosis. Warfarin is an indirect oral anticoagulant (OAC) and its anticoagulant effect is related to the inhibition of hepatic epoxide reductase, which results in reduced levels of active vitamin K. The association between warfarin and aortic stenosis is due to the inhibition of the vitamin-K-dependent protein Gla (MGP). In physiological conditions, MGP inhibits valve calcification by blocking the BMP2 pathway. When the BMP2 pathway is upregulated, it activates ALP, resulting in calcification of the aortic valve [83]. Koos et al. described an increase in documented valve calcium at CCT in patients treated with OAC [84]. In support of this finding, vitamin K supplementation demonstrated a reduction in the progression of valve calcification [85,86].

Magnesium contributes to regulating muscle contraction, immune responses, and bone formation and is also a cardiovascular protective factor. In vitro studies have described magnesium’s inhibitory action on the formation of hydroxyapatite crystals, thus blocking the calcification process of smooth muscle cells [87]. The recent ROADMAP study investigated the effects of magnesium supplementation and potassium reduction in valve calcification and chronic inflammation [88]. Iron and zinc homeostasis could also be the target of new evaluations for their involvement in the mechanism of disease progression.

In 2022, the European Atherosclerosis Society published a new consensus paper on Lp(a), summarizing the current knowledge on its causal association with atherosclerotic cardiovascular disease and aortic stenosis [89,90]. Increased Lp(a) is associated with an increased risk of aortic valve stenosis. A sub-study FOURIER has shown that PCSK9-i reduce Lp(a) levels and are associated with a reduction in new diagnoses of AS and valve replacement surgery [91,92].

Lp(a) is a low-density lipoprotein with an Apo(a) fraction covalently bound to its ApoB component. It has a diameter of <70 nm and can flow freely across the endothelial barrier, where it can be retained within the arterial wall to oxidize, trigger the process of lipid accumulation, and thus increase the risk of atherosclerotic cardiovascular disease (ASCVD), similar to LDL [93]. A measurement of Lp(a) should be considered at least once in each person’s lifetime, if available, to identify persons who have inherited an increased (>50 mg/dL) or extremely high level of Lp(a) (>180 mg/dL) and who therefore have a very high lifetime risk of ASCVD, roughly equivalent to the risk associated with HeFH (heterozygous familial hypercholesterolemia). Therefore, patients with significantly elevated Lp(a) should generally be treated with a target of <50 mg/dL (Table 2).

Previous studies have not demonstrated any benefit of statins on aortic valve progression. The SALTIRE study investigated the effects of intensive cholesterol reduction (with 80 mg atorvastatin) on the progression of aortic disease, describing no regression of aortic stenosis [102]. The randomized study simvastatin and ezetimibe in aortic stenosis (SEAS) described a reduction in ischemic cardiovascular events, but no difference for aortic valve events. About one-third of the patients in both groups experienced AVR: 28.3% in the simvastatin–ezetimibe group and 29.9% in the placebo group (HR, 1.00; 95% CI, 0.84 to 1.18; *p* = 0.97) [103]. Similarly, the result of the ASTRONOMER study described no effect of high-dose rosuvastatin on the progression of aortic stenosis [104]. On the other hand, statins have shown no effects or even an increase in Lp(a) with a 10% increase in hepatic Lp(a) secretion [94]. No serum Lp(a) changes were observed in response to treatment with ezetimibe and fibrates. The PCSK9-i, alirocumab, evolocumab, and inclisiran, are also an option for lowering Lp(a). Langsted et al. described that individuals carrying the PCSK9 R46L mutation have a reduced risk of ischemic cardiovascular disease and aortic stenosis. The mechanism is related to the loss of PCSK9 function, which is why PCSK9-i can lead to a reduction in the incidence and progression of aortic stenosis [105]. A meta-analysis showed that PCSK9-i reduced Lp(a) by 26% and improved cardiac outcomes [95].

CETP inhibitors (anacetrapib and evacetrapib) cause significant reductions in plasma Lp(a) levels through inhibition of its production [97,106]. One treatment option is daily niacin, which can reduce Lp(a) by 20% to 30%. However, niacin has not demonstrated an improvement in cardiovascular events, despite its beneficial effect on tLp(a) [96]. Hormonal drugs can reduce Lp(a) levels but their effect on cardiovascular outcomes appears controversial. Estrogens reduce Lp(a) levels, but their low safety profile makes their use in the cardiovascular field doubtful [107]. Testosterone replacement therapy (TRT) reduced Lp(a) levels but also HDL [108]. Further studies are needed to evaluate its use. Other therapies continue to emerge, but the key factor will be whether or not cardiac outcomes improve. Mipomersen, an antisense oligonucleotide (ASO) that inhibits apoB synthesis and the assembly of Lp(a), reduces ApoB levels by 31%, LDL-C levels by 32%, and Lp(a) levels by 30% [98]. Its therapeutic use is limited to patients with homozygous familial hypercholesterolemia due to hepatotoxicity (increased transaminases, hepatic steatosis, steatohepatitis, and risk of liver fibrosis and cirrhosis) [109]. 

Pelacarsen is an ASO conjugated with N-acetylgalactosamine (GalNAc), which reduces Lp(a) levels from 85 to 90%. GalNAc puts liver-specific targeting through binding to asialoglycoprotein, leading to the degradation of apo(a) mRNA [100]. The potent action of pelacarsen has been documented not only on Lp(a) but also on oxidized phospholipids (OxPL-apo(a) and OxPL-apoB). The mechanism seems to be associated with the reduction in Lp(a), which is a preferential lipoprotein transporter of OxPL. The specific targeting allows a higher potency of the drug with less systemic toxicity and lower dosage, as confirmed in early phase studies. Phase 3 of the lp(a)-HORIZON study enrolled 8323 patients with ASCVD and elevated baseline Lp(a) levels (>70 mg/dL) and evaluated the reductions in Lpa and MACE with pelacarsen.

Olpasiran is a GalNAc-conjugated siRNA that reduced Lp(a) by approximately 90%. GalNAc provides hepato-specific action and allows the double-stranded RNA molecule to enter the cell, where it dissociates. One antisense strand binds to the target mRNA sequence and degrades it. The other antisense strand binds to the RISC (RNA-induced silencing complex), forming a stable, recyclable complex so that inhibition of the target mRNA is repeated and sustained. Dosage and administration are therefore lower than ASOs. The phase 2 OCEAN(a) study described a reduction in Lp(a) in a dose-dependent manner in patients with ASCVD and Lp(a) > 150 nmol/L. The phase 3 study is ongoing and enrolling 6000 patients to evaluate the effect of olpasiran on cardiovascular outcomes and Lp(a) reduction.

Two other GalNAc-conjugated siRNAs that degrade apo(a) mRNA are currently evaluated in phase 1 studies. SLN360 reduced circulating Lp(a) by about 95% in cynomolgus monkeys. In phase 1, the drug was well tolerated in 32 patients, and a dose-dependent reduction in Lp(a) with a maximum reduction of approximately 97–98% was described [101]. The phase 1 study evaluating LY3819469 in 66 patients with elevated Lp(a) levels and increased cardiovascular risk was completed. The results are not yet available, but the subsequent phase 2 study has been registered. Further studies are needed to determine the safety and efficacy of these siRNAs. Ongoing clinical trials with these new therapies give us hope for effective therapies to reduce cardiovascular risk (Table 3). Unfortunately, these studies are not focused on aortic stenosis events and further research is needed to evaluate the Lp(a) inhibitor to slow or prevent the progression of calcific aortic stenosis.

## 5. Conclusions

Degenerative aortic stenosis is a disease of the elderly. Natural history is characterized by an unfavorable prognosis when symptoms begin. In recent years, echocardiographic assessments have been defined for the evaluation of stenosis and functional and structural alterations of the cardiac chambers. CCT is an essential tool for pre-procedural valuation and quantification of valve calcium. The future possibility of recognizing aortic stenosis by ECG, thanks to artificial intelligence, will change the diagnostic process. However, even if the diagnosis can be anticipated, there is currently no drug therapy approved for calcific/degenerative aortic stenosis. The ESC 2021 guidelines recommend TAVI for all patients with severe aortic stenosis of 75 years or older and for younger patients who are inoperable or at high surgical risk. The TAVI technique has evolved in recent years with the increased experience of operators and the introduction of new valves, and today, TAVI is considered a routine therapeutic strategy in many laboratories. However, the goal remains to slow or stop the progression of aortic valve degeneration. Initially, it was thought that the pathogenetic process could be linked to valve leaflet wear. With the discovery that the pathogenesis is similar to acute coronary syndromes, the pharmacological approach could become a therapeutic strategy. In particular, the pathogenetic mechanism is associated with dyslipidemia, inflammation, and calcification. Several molecules may interact by blocking the osteoblastic differentiation of VICs. Bisphosphonates and denosumab interact with inflammatory and osteogenetic mechanisms. However, randomized controlled trials are necessary to further investigate their role in aortic disease. End-stage renal disease correlates with vascular stiffening and valve calcification. Several drugs used in dialysis patients have been associated with a reduction in the progression of aortic stenosis, such as cinacalcet, vitamin K supplementation, magnesium, and potassium while warfarin has shown a negative prognostic effect. High blood Lp(a) levels are correlated with a high risk of cardiovascular and aortic events. Currently, available antilipidemic drugs cause a low reduction in circulating Lp(a) levels. New molecules are currently involved in clinical trials and have been shown to reduce Lp(a) levels by 85–90%. The phase 3 HORIZON study will investigate the cardiovascular outcomes of pelacarsen, an ASO that degrades apo(a) mRNA and reduces blood levels of Lp(a) and oxidized lipids. The phase 3 OCEAN(a) study will evaluate olpasiran, a siRNA capable of degrading apo(a) mRNA and prolonging its action by inhibiting RISC. Two other siRNAs, SLN360 and LY3819469, are currently involved in dose, efficacy, and safety studies. Unfortunately, these studies will not assess the progression of aortic stenosis or aortic events, and further clinical studies should be specifically performed to evaluate the pharmacological-induced Lp(a) reduction in this clinical setting.

## Figures and Tables

**Table 1:** Echocardiographic assessment of aortic stenosis [61].

	Mild	Moderate	Severe
AV Peak Velocity	2–2.9 m/s	3–3.9 m/s	>4 m/s
AV Mean Gradient	<20 mmHg	20–39 mmHg	>40 mmHg
AVA	>1.5 cm^2^	1.4–1 cm^2^	<1 cm^2^ (AVA/BSA < 0.6 cm^2^/m^2^)

AV, aortic valve; AVA, aortic valve area.

**Table 2:** Effect of anti-lipid drugs on Lp(a) blood concentration.

Drugs	Mechanism of Action	Effect on Lp(a)
Statins [94]	Increase Lp(a) production from hepatocytes	Increase ~10%
Ezetimibe	None	No effect
Fibrates	None	No effect
PCSK9i [95]	Increase Lp(a) catabolism and decreased Lp(a) production	Decrease ~25%
Niacin [96]	Lpa gene transcription downregulation is mainly due to the reduction in intracellular CAMP levels	Decrease ~20%
CETPi [97]	Inhibition of transferring cholesteryl esters from HDL to Apolipoprotein B containing particles	Decrease ~30%
Mipomersen [98,99]	Apolipoprotein B-100 synthesis inhibition	Decrease ~25%
Lipoprotein apheresis	Selective Apoliprotein B-100 containing clearance from plasma	Decrease ~60%
ASOs and siRNA [100,101]	Apolipoprotein(A) synthesis inhibition	Decrease ~85–90%

Lp(a), lipoprotein(a); CAMP, cyclic adenosine monophosphate; HDL, high-density lipoprotein; ASOs, antisense oligonucleotides; siRNA, small interfering RNAs.

**Table 3:** New drugs and clinical trials to reduce Lp(a).

Drug and Type	Clinical Trial	Population	Treatment	Results
PELACARSEN GalNAc-conjugated ASO	*NCT02414594* (completed) [110]	*n* = 58 Healthy subjects Lp(a) ≥ 30 mg/dL (≥75 nmol/L)	Single ascending dose: 10 mg, 20 mg, 40 mg, 80 mg, or 120 mg; multiple ascending doses: 10 mg, 20 mg, or 40 mg.	Lp(a) reduction: single dose: 26–85%; multiple doses: 66–92%.
	*NCT03070782* (completed) [111]	*n* = 286 Lp(a) ≥ 60 mg/dL (≥150 nmol/L) CVD is defined as documented coronary artery disease, stroke, or peripheral artery disease	20 mg once a week; 20 mg every 2 weeks; 20 mg, 40 mg, or 60 mg every 4 weeks.	Lp(a) reduction: 35–80%.
	Lp(a)-HORIZON trial *NCT04023552* (ongoing, expected to be completed in 2025) [112]	*n* = 8323 Lp(a) ≥ 70 mg/dL (175 nmol/L) CVD is defined as symptomatic peripheral artery disease, prior myocardial infarction or ischemic stroke before the screening visit (10 years to 3 months)	80 mg every 4 weeks.	No data are available.
OLPASIRAN GalNAc-conjugated siRNA	*NCT04987320* (completed) [113]	*n* = 27 Healthy subjects Lp(a) ≥ 28 mg/dL (≥70 nmol/L)	3 mg, 9 mg, 75 mg, or 225 mg (single dose)	Lp(a) reduction: 56–99%.
	*NCT03626662* (completed) [114]	*n* = 24 Healthy subjects Lp(a) 28–79 mg/dL (70–199 nmol/L) *n* = 40 Healthy subjects Lp(a) ≥ 80 mg/dL (≥200 nmol/L)	3 mg, 9 mg, 30 mg, 75 mg, or 225 mg (single dose).	Lp(a) reduction: 71–97%.
	*NCT04270760* (completed) [115,116]	*n* = 281 Healthy subjects Lp(a) > 60 mg/dL (>150 nmol/L) Evidence of ASCVD	10 mg, 75 mg, and 225 mg every 12 weeks or 225 mg every 24 weeks.	Lp(a) reduction: 70.5–100.5%.
	OCEAN(a) trial *NCT05581303* (ongoing, expected to be completed in 2026)	*n* = 6000 (target)Lp(a) ≥ 80 mg/dL (≥200 nmol/L) History of ASCVD defined as myocardial infarction and/or percutaneous coronary revascularization and at least 1 additional risk factor	Single dose every 12 weeks.	No data are available.
SLN360 GalNAc-conjugated siRNA	*NCT04606602* [101]	*n* = 32 Lp(a) ≥ 60 mg/dL (≥150 nmol/L) Confirmed history of stable ASCVD	30 mg, 100 mg, 300 mg, or 600 mg (single dose).	Lp(a) reduction: 46–98%.
	*NCT05537571*	*n* = 160 (target) Lp(a) ≥ 50 mg/dL (≥125 nmol/L) high risk of ASCVD events	Three different doses.	No data are available.
LY3819469 GalNAc-conjugated siRNA	*NCT04914546*	*n* = 66 (target) Part A: healthy subjects, high Lp(a) levels Part B: Japanese participants	No data are available.	No data are available.

GalNAC, N-acetylgalactosamine; Lp(a), lipoprotein(a); CVD, cardiovascular disease; ASCVD, atherosclerotic cardiovascular disease.

## Data Availability

No new data were created or analyzed in this study. Data sharing is not applicable to this article.
